# Hamman’s Syndrome in the Context of Influenza A and Regular Vaping in a Young Adult

**DOI:** 10.7759/cureus.94083

**Published:** 2025-10-07

**Authors:** Shyam Gautam, Sony Manandhar, Robin Sharma, Trishita Datta, Oluwapelumi O Akintade

**Affiliations:** 1 Department of Medicine for Older People, Stepping Hill Hospital, Stockport NHS Foundation Trust, Stockport, GBR

**Keywords:** hamman’s syndrome, influenza a, spontaneous pneumomediastinum, subcutaneous emphysema, vaping

## Abstract

Spontaneous pneumomediastinum, also called Hamman’s syndrome, is an uncommon condition that results from alveolar rupture with air tracking into the mediastinum. It is usually triggered by events that raise intrathoracic pressure, but because of its rarity, it is often overlooked and may initially be confused with more serious conditions. The prognosis is generally excellent, as most cases resolve without intervention. We describe the case of a woman in her early 20s, with a history of regular e-cigarette use, who developed acute chest pain, shortness of breath, vomiting, and difficulty swallowing during an influenza A infection. Initial chest radiography demonstrated subcutaneous emphysema, pneumomediastinum, and a small left apical pneumothorax, raising concern for Boerhaave syndrome. CT confirmed pneumomediastinum without evidence of oesophageal perforation, which was further excluded by a water-soluble contrast swallow study. A viral screen was positive for influenza A. She was managed conservatively with broad-spectrum antimicrobials, oseltamivir, nil by mouth, and parenteral nutrition until Boerhaave syndrome was excluded, followed by gradual reintroduction of diet and supportive therapy. Her symptoms resolved completely. This case highlights the potential synergistic effect of viral respiratory infection and vaping in precipitating Hamman’s syndrome in otherwise healthy young adults and underscores the importance of distinguishing this benign entity from life-threatening differential diagnoses such as oesophageal rupture.

## Introduction

The term pneumomediastinum (PM) refers to the presence of free air in the mediastinum, while surgical emphysema describes the same phenomenon in subcutaneous tissue. Both are characterised by air leakage from the alveolus into a specific anatomical zone, such as the mediastinum or subcutaneous tissue [1]. Classically, PM can be of two forms: secondary, where there is a defined aetiology, and idiopathic, where the cause is not obvious - the latter also known as spontaneous PM (SPM) [2]. SPM and subcutaneous emphysema are often referred to collectively as Hamman’s syndrome.

SPM is commonly associated with the Valsalva manoeuvre, forceful coughing, excessive vomiting, and, in rare cases, labour. Classical pathophysiology involves the rupture of the terminal alveolus secondary to high intrathoracic pressure, which can be caused by the above, leading to air accumulation in the interstitium, and then dissecting towards the mediastinum, known as the Macklin effect [3,4]. Presentation of SPM includes dyspnoea, cough, neck pain, dysphagia, and, in certain individuals, Hamman’s sign - a bubbling sound that synchronises with the heartbeat [2].

SPM is a rare clinical entity, with an incidence among adult inpatients reported to range from 0.001% to 0.01%; this variability is largely explained by differences in diagnostic criteria, imaging methods, and institutional practices. Most reported cases involve individuals between 18 and 25 years of age, with males comprising approximately 73.1% of the patient population [5].

Data from a nationwide health survey conducted in 2021 show that 4.5% of adults aged over 18 years use e-cigarettes, with the highest proportion (11%) among those aged 18-24 years [6]. The growing use of e-cigarettes and vaping among younger individuals predisposes them to SPM through the Valsalva manoeuvre [7].

Persisting vomiting, which can happen in influenza, may increase the risk of SPM in young patients with a background of vaping. To the best of our knowledge, multiple cases of SPM linked to vaping have been reported, but only two cases have been documented involving both vaping and influenza infection [1,8]. This is a case of a young female in her 20s who tested positive for influenza A and had a history of e-cigarette use, later diagnosed with Hamman’s syndrome.

## Case presentation

A 20-year-old patient presented with a five-day history of coryzal symptoms, myalgia, and repeated vomiting. Two days before presentation, she developed a sore throat, worsening vomiting, inability to tolerate oral intake, difficulty swallowing, and sharp epigastric chest pain. She had been started on oral antibiotics in primary care for presumed acute tonsillitis. She reported daily use of e-cigarettes for 1.5 years, along with occasional recreational inhalation of marijuana, with no significant past medical history. On examination, she was alert, oriented, and haemodynamically stable, with a Glasgow Coma Scale score of 15/15. Respiratory assessment revealed palpable crepitus over the supraclavicular region, mild shortness of breath with increased respiratory effort, and possible crackles, but no reduction in breath sounds. The abdominal examination showed mild epigastric tenderness without guarding.

Initial laboratory investigations are summarised in Table 1. Haematology showed mild anaemia, an elevated inflammatory marker, and normal electrolytes. Renal function was impaired, consistent with stage 2 acute kidney injury. Liver function tests and serum proteins were normal. Venous blood gas analysis demonstrated alkalosis with low bicarbonate, hypoxemia, hypocapnia, and elevated lactate.

**Table 1:** Initial laboratory results with reference ranges CRP, C-reactive protein; eGFR, Estimated glomerular filtration rate; HCO₃⁻, Bicarbonate; PCO₂, Partial pressure of carbon dioxide; PO₂, Partial pressure of oxygen; VBG, Venous blood gas; WCC, White cell count

Test	Result	Reference range
WCC	10.6 × 10⁹/L	4.0-11.0 × 10⁹/L
Haemoglobin	115 g/L	115-165 g/L
CRP	33.1 mg/L	0-10 mg/L
Sodium	140 mmol/L	135-145 mmol/L
Potassium	3.5 mmol/L	3.5-5.3 mmol/L
Urea	17.1 mmol/L	2.5-7.8 mmol/L
Creatinine	118 µmol/L	62-115 µmol/L
eGFR	52 mL/min/1.73 m²	>60 mL/min/1.73 m²
VBG pH	7.5	7.35-7.45
PO₂ (venous)	2.9 kPa	4.5-6.0 kPa (venous)
PCO₂	3.1 kPa	4.5-6.0 kPa
HCO₃⁻	20.7 mmol/L	22-28 mmol/L
Lactate	3.5 mmol/L	<2.0 mmol/L

**Figure 1:**
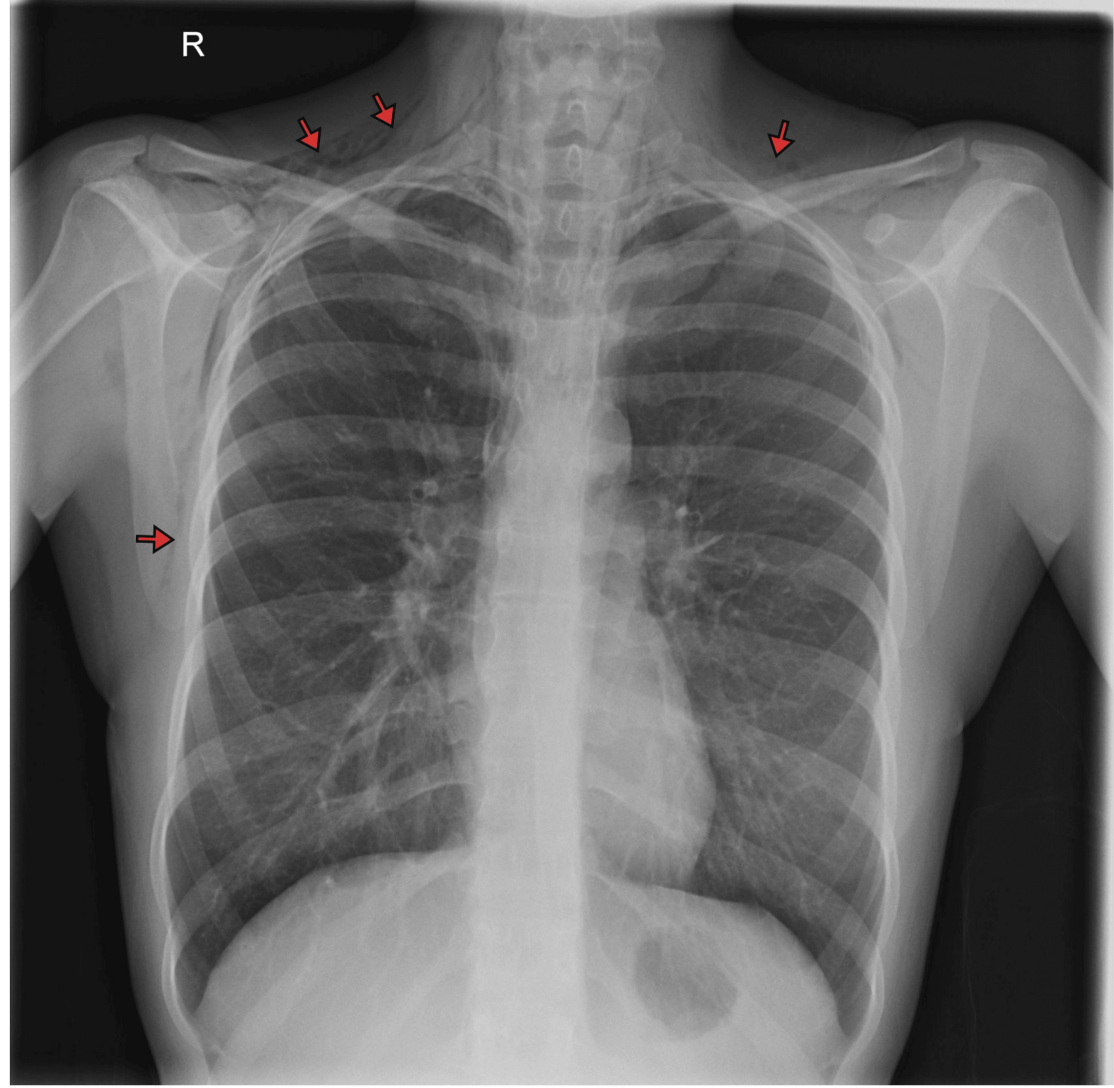
Chest radiograph showing subcutaneous emphysema This posteroanterior chest radiograph demonstrates extensive subcutaneous emphysema, visible as radiolucent streaks of gas (highlighted by red arrows) within the soft tissues of the supraclavicular region, lower neck, and axilla.

**Figure 2:**
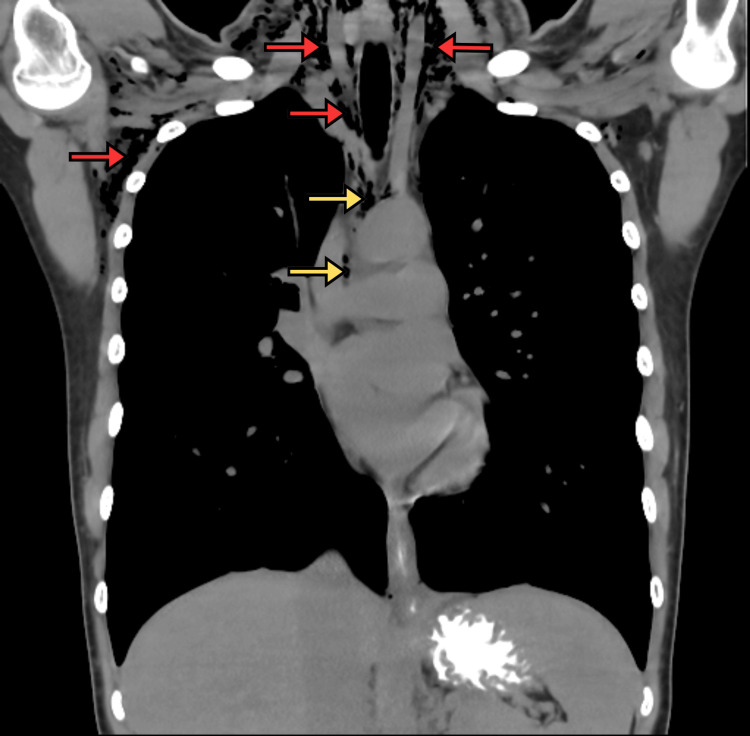
CT chest showing subcutaneous emphysema in the soft tissues of the neck and axilla, with pneumomediastinum Coronal CT imaging of the thorax reveals extensive subcutaneous emphysema, visible as radiolucent air pockets (red arrows) within the cervical, supraclavicular, and axillary soft tissues. Air is also observed within the mediastinum (yellow arrows), consistent with pneumomediastinum.

A chest radiograph on day one (Figure 1) revealed a small left apical pneumothorax and extensive subcutaneous emphysema in the soft tissues of the neck and axilla, with air tracking into the mediastinum (PM), which was more evident on CT of the thorax (Figure 2).

**Figure 3:**
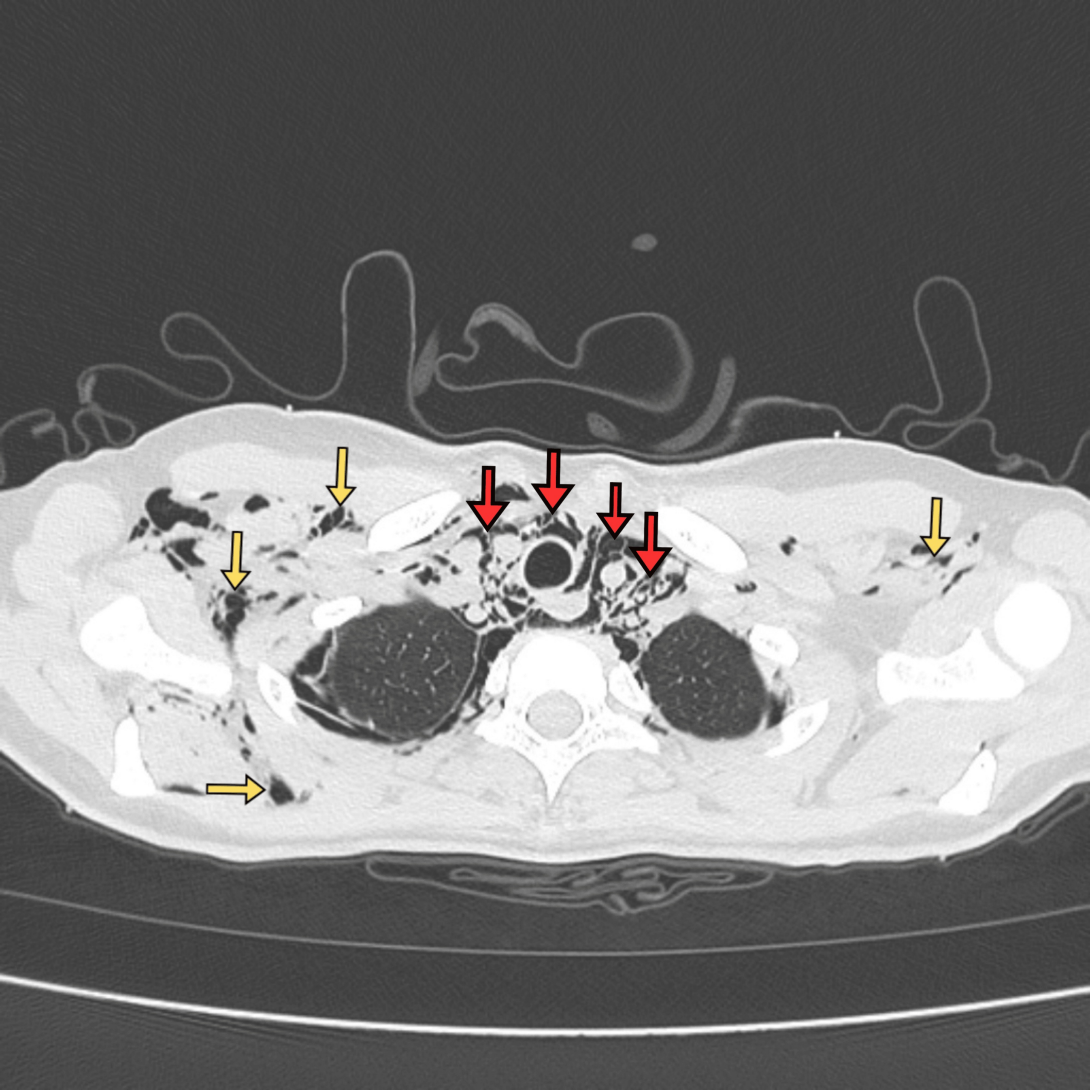
Axial CT chest Axial CT chest showing subcutaneous emphysema (yellow arrows indicate air pockets in the subcutaneous tissues of the neck and axilla) and pneumomediastinum (red arrows highlight mediastinal air around the paratracheal region)

CT of the thorax (Figure 3) confirmed PM with small gas volumes in the bilateral oblique fissures, without a large pneumothorax. A respiratory viral screen was positive for influenza A. Initially, Boerhaave syndrome was considered, given the history of vomiting and the presence of mediastinal air; however, this diagnosis was excluded by CT imaging and contrast swallow studies, which demonstrated no extraluminal contrast leak to suggest oesophageal rupture. The final diagnosis of Hamman’s syndrome (SPM) was made based on clinical and radiological findings in the absence of trauma or oesophageal perforation.

**Figure 4:**
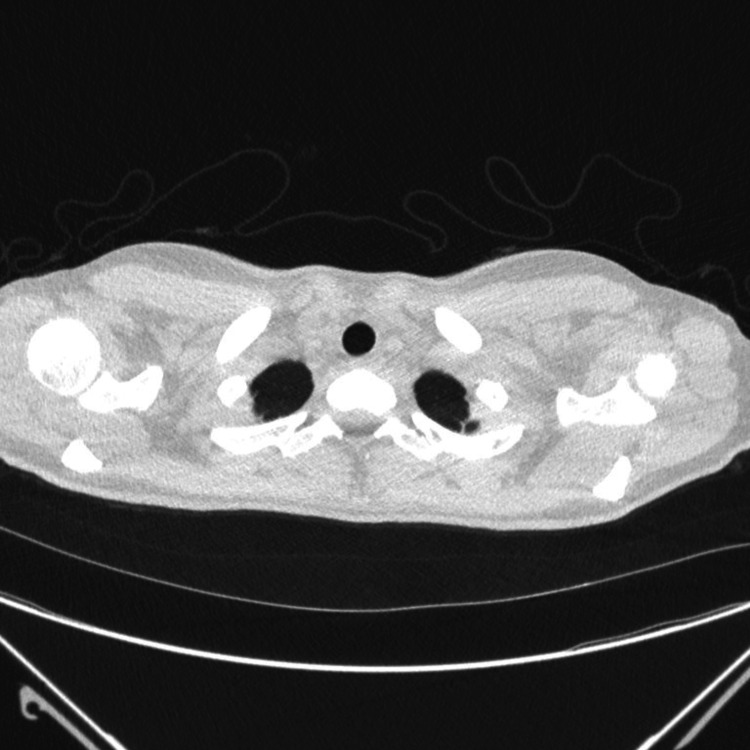
Axial CT chest at outpatient follow-up The scan demonstrates complete resolution of the previously noted subcutaneous emphysema and pneumomediastinum.

The patient was managed conservatively under joint medical and surgical care. She remained nil by mouth for seven days with parenteral nutrition support, started on broad-spectrum antibiotics for five days, and received oseltamivir for influenza A. Analgesia and supplemental oxygen were provided. Oral intake was reintroduced on day eight, starting with clear fluids and gradually advancing to a full diet as tolerated. The patient’s symptoms resolved within several days. She was discharged after 10 days with advice on vaping cessation and avoidance of activities that increase intrathoracic pressure. At outpatient review, high-resolution CT confirmed complete resolution of PM, pneumothorax, and subcutaneous emphysema (Figure 4).

## Discussion

Hamman's syndrome, also known as Macklin’s syndrome, was named after the clinician Louis Hamman. It is an uncommon yet important differential diagnosis to consider in a young patient with chest pain and dyspnoea. The pathophysiology of Hamman's syndrome is explained by the dissection of air in the bronchovascular sheath, when elevated intra-alveolar pressure, low peri-alveolar pressure, or a combination of both causes alveolar rupture [9]. This is known as Macklin’s effect. This can be triggered by Valsalva manoeuvres, coughing, vomiting, or inhalation of substances such as e-cigarettes, marijuana, and cocaine.

Hamman's syndrome can present with chest pain, dyspnoea, neck pain, and subcutaneous emphysema. A distinctive clinical finding on auscultation is the presence of crackles that are synchronised with the heartbeat, referred to as Hamman’s sign. While this sign is highly suggestive of SPM, it is observed in fewer than half of all cases [10].

Vaping may induce alveolar injury through both mechanical and chemical mechanisms. Deep inhalation and frequent Valsalva-like manoeuvres associated with vaping can increase intrathoracic pressure, predisposing to alveolar rupture. Whereas in marijuana users, smoking techniques and marijuana-induced vomiting are the precipitating factors for SPM [11]. Forceful inhalation through a high-resistance vaping device may generate substantial negative intrathoracic pressure, predisposing to spontaneous pneumothorax through the mechanism of the Müller manoeuvre [12].

To date, only four cases of spontaneous pneumothorax associated with marijuana vaping have been reported in patients aged 18-35 years [12], and three cases of SPM related to e-vaping have been documented in patients aged 20-26 years [5,13,14]. However, as shown in Table 2, only two reported cases have been attributed to concurrent influenza infection and e-cigarette use (one case with influenza A and another with influenza B) [1,8].

**Table 2:** Summary of cases attributed to influenza infection in association with vaping/e-cigarette use

S.N.	Age and sex	Type of influenza	Association	Presentation	Treatment	Outcome	Location	Any complication associated with the disease	Reference
1	20 years, female	Influenza A	e-cigarette use, does not smoke tobacco, and reports marijuana use two weeks before	Shortness of breath, cough, wheeze, multiple episodes of non-bloody, non-bilious emesis, and flu-like symptoms	Conservative, symptomatic management, steroids, oseltamivir, and F/U in chest clinic	Complete resolution	USA	Nil	[1]
2	22 years, male	Influenza B	Daily vaping/e-cigarette	Cough, shortness of breath, low-grade fever, vomiting, and flu-like symptoms	Supplemental oxygen, oseltamivir, antibiotics, and counselling against vaping	Resolution	USA	Bilateral pneumonia	[8]

In this case, influenza A infection likely precipitated SPM in the absence of trauma or underlying airway disease, with cough and vomiting episodes generating sufficient intrathoracic pressure to induce alveolar rupture and air dissection along perivascular sheaths. The patient’s history of vaping, in combination with these factors, may have further contributed to the development of Hamman’s syndrome [1]. Differentiating SPM from more serious conditions, particularly oesophageal rupture (Boerhaave syndrome), can be challenging in primary care settings. Unlike the typically benign course of SPM, oesophageal rupture can lead to mediastinitis and is potentially life-threatening [15]. On the other hand, unfamiliarity with the entity can lead to unnecessary diagnostic tests and inappropriate treatment [16].

Imaging, particularly chest X-ray and CT, plays a central role in diagnosis. CT is more sensitive and can help distinguish SPM from more emergent conditions.

Management is usually conservative, with supplemental oxygen, analgesia, and rest. Routine observation and follow-up are recommended for all [3]. Lifestyle counselling, particularly regarding cessation of vaping and marijuana use, is essential to prevent recurrence. 

## Conclusions

This report describes a case of Hamman’s syndrome in a previously healthy young female with a history of Influenza A infection and vaping. Episodes of severe coughing and vomiting likely triggered alveolar rupture due to elevated intrathoracic pressure, compounded by pre-existing alveolar vulnerability from inhalational exposure. This case emphasises the importance of a thorough differential diagnosis, early imaging, and a multidisciplinary approach to avoid unnecessary interventions and to provide appropriate supportive care. This case also highlights the role of increased intrathoracic pressure from vomiting and inhalational injury (smoking marijuana and vaping) as potential triggers for Hamman’s syndrome in young individuals. Lifestyle counselling is key to preventing recurrence, particularly in patients who vape or engage in similar risk behaviours. Given the paucity of reported cases of SPM associated with both influenza A infection and vaping, the interplay between these factors remains poorly understood. Further studies are warranted to clarify their potential combined role in the pathogenesis of SPM.
